# Cytomegalovirus retinitis in patients of non-Hodgkin’s lymphoma: clinical presentations and outcomes

**DOI:** 10.1186/s12348-021-00257-z

**Published:** 2021-10-06

**Authors:** Subhakar Reddy, Mudit Tyagi, Shashwat Behera, Rajeev R. Pappuru, Vivek P. Dave, Soumyava Basu, Hitesh Agrawal

**Affiliations:** 1 Uveitis and Ocular Immunology Services, LV Prasad Eye Institute, Hyderabad, -500034 India; 2 Smt Kanuri Santhamma Center for Vitreo-Retinal Diseases, L. V. Prasad Eye Institute, Hyderabad, -500034 India

**Keywords:** Cytomegalovirus retinitis, Non-Hodgkin’s lymphoma, Chemotherapy, Ganciclovir

## Abstract

**Background:**

Cytomegalovirus (CMV) retinitis in patients with Non-Hodgkin’s Lymphoma (NHL) can occur even in the presence of high CD 4 counts and can behave differently when compared to CMV retinitis in human immunodeficiency (HIV) patients. It, therefore, becomes important to understand its varied presentations and the challenges in management of these cases. The aim of this study was to analyse the various patterns of presentations and outcomes of CMV Retinitis in patients with NHL.

**Study design:**

A retrospective chart review of seven eyes of four patients of NHL presenting with CMV retinitis between June 2017 and May 2020 was done.

**Methods:**

Clinical patterns of CMV Retinitis, CD4 counts at the time of presentation and the duration of treatment along with recurrences and time for recurrence of retinitis were assessed.

**Results:**

Granular or indolent retinitis (6 out of 7 eyes) was the commonest form of CMV retinitis in patients of NHL. Three patients had a presenting CD4 count above 150 cells/mm^3^ and none of them were below 50 cells/mm^3^. Floaters were the commonest presenting complaint. All patients had vitritis and majority of the patients (3 out of 4) had anterior chamber (AC) inflammation. Two out of the 4 patients had a recurrence (mean time 33.8 days) after stopping the maintenance phase of ganciclovir and one patient had significant myelosuppression related to oral valganciclovir which required discontinuation of the drug.

**Conclusion:**

CMV retinitis in NHL patients is usually of an indolent or granular type and can occur even in the presence of high CD4 counts as compared to patients with HIV. These patients may require a long term maintenance in view of frequent recurrences after discontinuation of treatment.

## Background

Cytomegalovirus (CMV) is a double-stranded DNA virus belonging to the family of Herpesviridae [1]. The global prevalence of CMV is believed to be around 83% in the general population [2]. After entering the body, CMV is initially detected by the innate immune system via Toll-like receptors and Natural killer (NK) cells initiating a humoral response [3]. However, long-term control of CMV is predominantly through cell-mediated immunity [4, 5]. CMV remains latent in peripheral blood leukocytes and bone marrow cells of hosts. However, it can get reactivated in immunocompromised individuals resulting in several opportunistic infections. The most common ocular features of CMV infection include hemorrhagic retinitis, vasculitis, and retinal necrosis. CMV retinitis is the most common ocular opportunistic infection and the leading cause of ocular morbidity associated with Acquired immunodeficiency syndrome (AIDS) [6]. However, apart from AIDS, even malignancies and chemotherapies that target T cells are known to increase the risk of CMV disease [3]. Non-Hodgkin’s Lymphoma (NHL) is a reticuloendothelial cell carcinoma which needs multiple sessions of chemotherapy or radiotherapy. CMV retinitis in patients of NHL who have received prior chemotherapy or radiotherapy has been sparsely reported and most of the literature is confined to few case reports and needs to be studied in detail. This study aims to describe the clinical presentations and outcomes of CMV retinitis in four patients of NHL who had presented to the Uveitis services of our institute.

## Methods

A retrospective chart review of all patients with NHL presenting with CMV retinitis between June 2015 and May 2020 was done. Seven eyes of four patients were included in the study.

The study was evaluated and approved by the Institutional Ethics Committee of LV Prasad Eye Institute (LEC-BHR-R-08-20-490). Informed written consent was obtained from all the patients before including in the study. All the cases were analysed for clinical presentations, Cluster of Differentiation (CD4) counts at the time of presentation, clinical outcomes, and response to treatment. All the patients had undergone a complete ophthalmic evaluation including fundus photographs. The anterior chamber reaction was graded as per the guidelines of the Standardization of Uveitis Nomenclature (SUN) group [7] and the vitreous inflammation was graded based on Nussenblatt/NEI methodology [8].

### Case 1

**Fig. 1:**
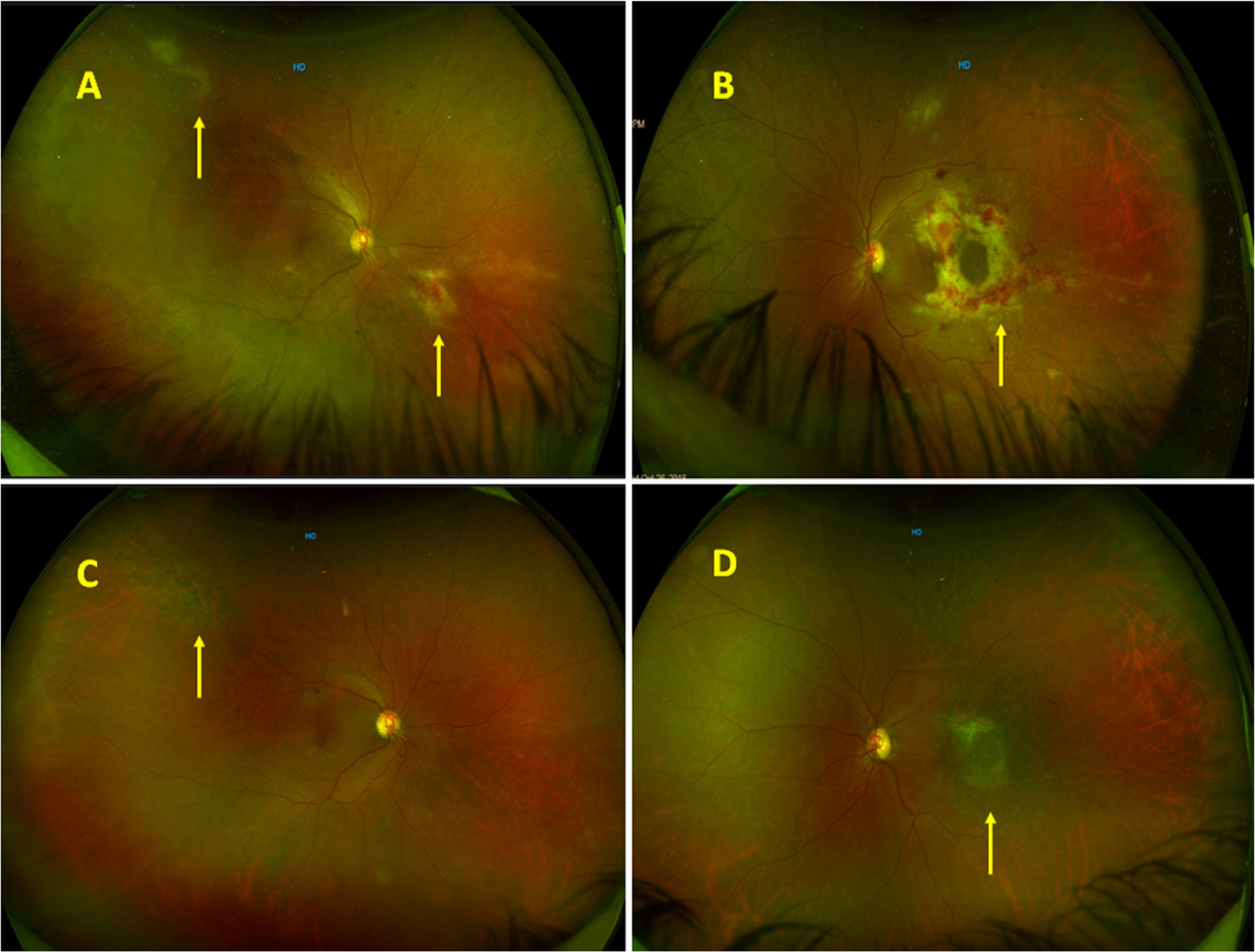
**A** Fundus photographs of the right eye showing fulminant retinitis inferonasal to disc and granular retinitis lesion in superotemporal periphery (yellow arrows). **B** Fundus photographs of the left eye showing fulminant retinitis at the macula (yellow arrow) and granular retinitis superiorly. **C,D** Fundus photographs of right and left eye respectively showing completely healed retinitis (yellow arrows) at last follow-up with mild optic disc pallor and epiretinal membrane in the left eye

A 59-year-old female presented with complaints of floaters in both eyes for 3 months after an episode of chickenpox. She was a known case of marginal zone Lymphoma and had received six cycles of chemotherapy. Her disease was under remission with targeted chemotherapy with oral Ibrutinib for 6 months. At the time of presentation, her best-corrected visual acuity (BCVA) was 20/40 in both eyes. Intraocular pressure measured by Goldmann applanation tonometer in the right and left eye was 15 and 14 mm of Hg respectively. Anterior segment examination showed 1+ cells in both eyes. Fundus examination of the right eye showed vitritis 1+ in both eyes along with fulminant retinitis inferonasal to disc and granular retinitis lesion in superotemporal periphery (Fig. 1A). The left eye showed fulminant retinitis at macula along with an area of granular retinitis superiorly (Fig. 1B). A diagnosis of CMV retinitis in both eyes was made and a subsequent vitreous biopsy was done along with intravitreal ganciclovir injection. PCR of vitreous sample was positive of CMV DNA and was negative for Varicella Zoster Virus, Herpes simplex and Toxoplasma. Her CD4 counts were found to be 109 cells/mm^3^ and she was seronegative for HIV. Initially, it was planned to start her on oral Valganciclovir. However she also had thrombocytopenia (60,000/μl) and leucopenia (1800 cells/ μl) and therefore she was started on intravitreal ganciclovir (2.5 mg/0.1 ml). After 10 intravitreal ganciclovir injections, the retinitis had resolved. Her CD4 counts at this point were 139 cells/mm^3^. After 3 months of treatment with intravitreal injections, her intravitreal injections were discontinued in view of resolved retinitis. However, she had a recurrence after 16 days of stopping intravitreal injections. Her CD4 count at this point of time was 130 cells/ mm^3^. She was again started on intravitreal ganciclovir and subsequently she was shifted to oral valganciclovir since her leucocyte counts and platelets had stabilized. The maintenance dose of valganciclovir 900 mg once daily was continued for 2 more months. However, after stopping treatment, she had a second recurrence again in the right eye after 32 days. The CD4 counts at the time of recurrence were 219 cells/ mm^3^. She was restarted on oral valganciclovir. The BCVA in the right and left eye was 20/100 and 20/400 respectively at the time of her last follow-up (22 months). Fundus evaluation showed disc pallor in both the eyes along with an epiretinal membrane (ERM) in the left eye (Fig. 1C,D).

### Case 2

**Fig. 2:**
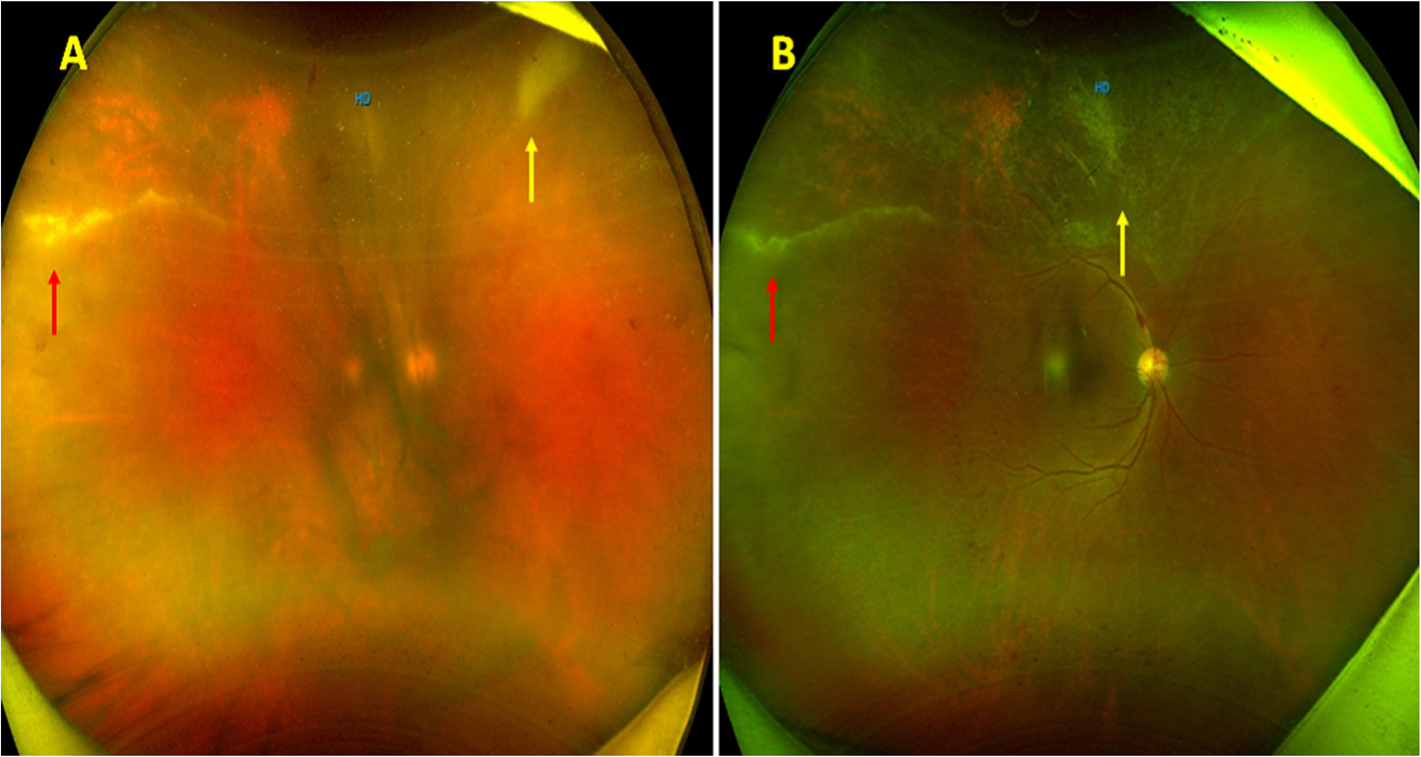
**A** Fundus photographs of the right eye showing dense vitritis, granular retinitis (yellow arrow) superiorly with retinal vasculitis in superotemporal periphery (red arrow). **B** Fundus photographs at follow-up showing healed retinal vasculitis (red arrow) and resolved retinitis (yellow arrow)

A 63-year-old male presented to the Uvea services with complaints of blurring of vision and floaters in his right eye for 6 months. He was a known case of NHL and had received 12 cycles of chemotherapy over the previous 6 months and his lymphoma was under remission. He was initially diagnosed as having intermediate uveitis and had been started on oral steroids elsewhere. At the time of presentation, his BCVA was 20/100 in the right eye and 20/30 in the left eye. Anterior segment examination was essentially normal in both eyes except for lenticular sclerosis. Intraocular pressure in the right and left eye was 16 and 12 mm of Hg respectively. Fundus examination of the right eye (Fig. 2A) showed hazy media due to grade 3+ vitritis along with ground glass granular retinitis lesion superiorly and confluent retinal vasculitis in superotemporal quadrant. The left eye fundus was essentially normal. A diagnosis of right eye viral retinitis was made and a subsequent vitreous biopsy was done along with intravitreal ganciclovir injection. His vitreous sample was sent for microbiological analysis. PCR was positive for CMV DNA and negative for VZV, HSV and Toxoplasma. His CD4 counts were found to be 278 cells/mm^3^. The CD4:CD8 ratio was 1.68:1. His HIV screening came out to be negative. He was started on Valganciclovir (900 mg twice daily for 2 weeks) and currently he is on a maintenance dose of Valganciclovir 900 mg once daily. At the last follow-up (6 months) his vision had improved to 20/30 in the right eye with healed retinitis and vasculitis (Fig. 2B).

### Case 3

**Fig. 3:**
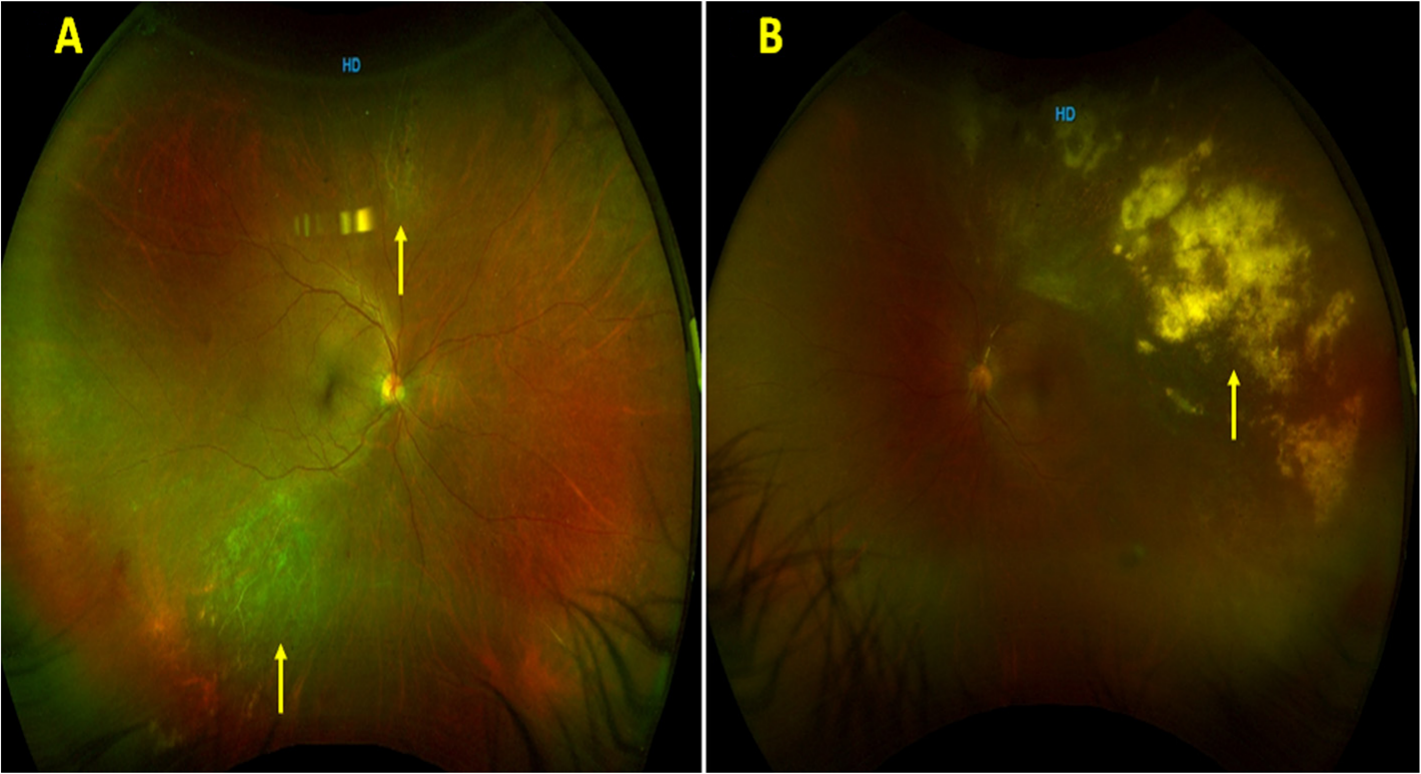
**A** Fundus photographs of the right eye showing granular retinitis inferotemporally and superiorly (yellow arrow). **B** Fundus photographs of the left eye showing granular retinitis (yellow arrow) superotemporally with retinal vasculitis superior to disc

A 54-year-old male patient presented to the emergency clinic with complaints of floaters in both eyes for 4 months. He was a known case of Diffuse Large B cell lymphoma and had received 25 sessions of radiotherapy and 6 cycles of chemotherapy, which had been completed 3 months before presentation to our clinic. A complete ophthalmic evaluation was done. His BCVA was 20/20 in the right eye and 20/40 in the left eye. Anterior segment examination showed anterior chamber (AC) cells 2+ in both eyes. IOP in the right and left eyes were 11 and 12 mm of Hg respectively. He had vitritis 1+ in both eyes. Fundus examination of the right eye (Fig. 3A) showed perivasculitis with granular retinitis lesion superiorly, inferotemporally, and left eye fundus (Fig. 3B) showed large retinitis lesion superiorly and temporally with retinal vasculitis superior to disc. So, a diagnosis of both eye CMV retinitis was made and a subsequent vitreous biopsy was done along with intravitreal ganciclovir injection. His vitreous sample was sent for PCR. Conventional PCR was negative for all viruses CMV, VZV, HSV, and also for Toxoplasma. CD4 counts were found to be 180 cells/mm^3^ and he was seronegative for HIV. He was initially started on a twice-weekly regimen of intravitreal ganciclovir injections. The retinitis lesions started to resolve with intravitreal ganciclovir injections. He was subsequently shifted to oral valganciclovir after the induction phase and then maintained on once daily dose of 900 mg of oral valganciclovir for 3 months. Subsequently valganciclovir was discontinued and the BCVA in the right and left eye had improved to 20/20 and 20/30 at the time of his last follow-up (4 months) with completely regressed retinitis. The patient was subsequently lost to follow-up.

### Case 4

**Fig. 4:**
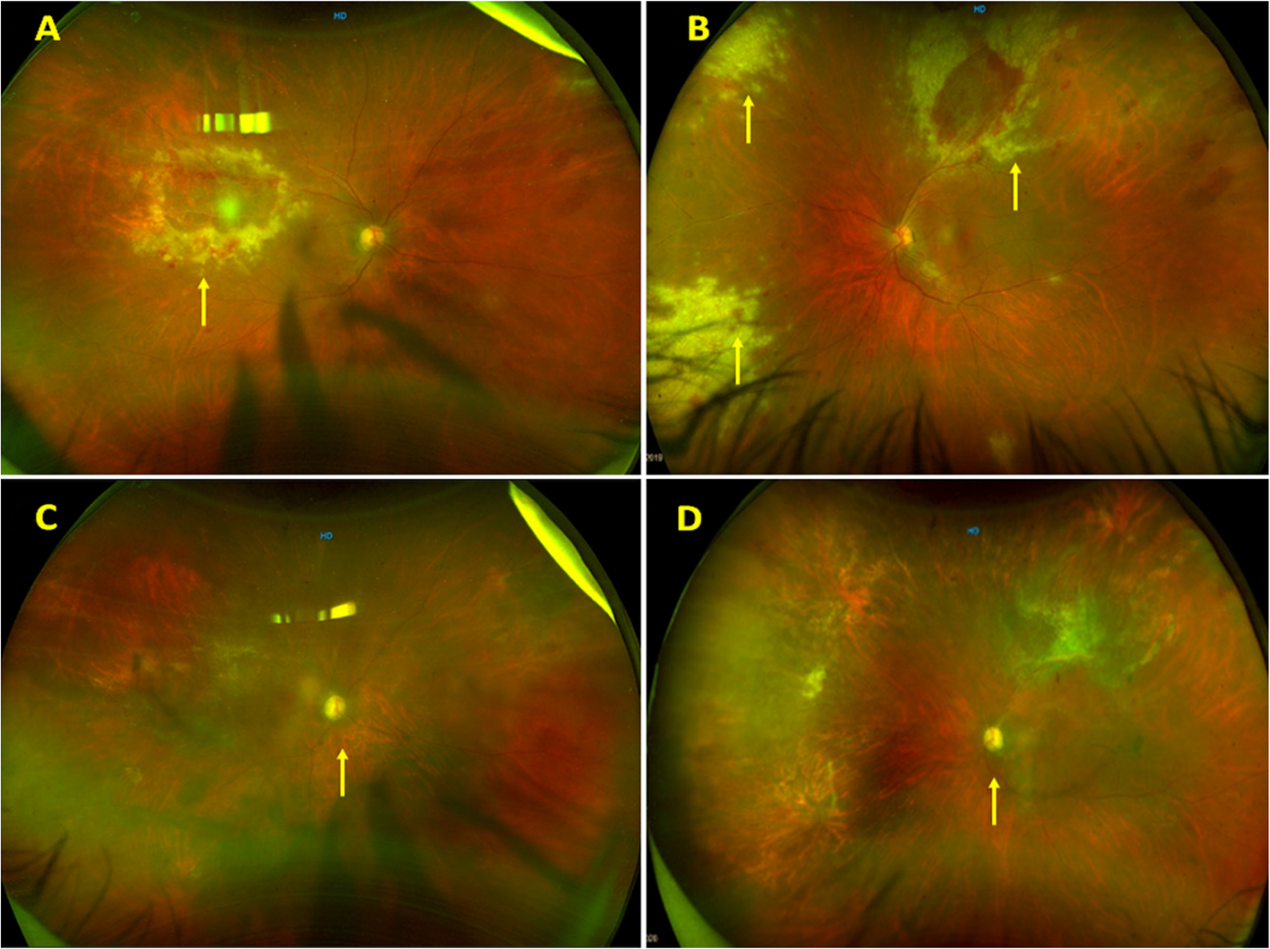
**A** and **B**: Fundus photographs of the right and left eyes respectively showing granular retinitis superotemporal to the macula in the right eye and multiple retinitis patches in the left eye (yellow arrow). **C** and **D**: Fundus photographs of the right and left eyes respectively showing resolved retinitis with a pale optic disc (yellow arrow)

A 58-year-old male patient presented with complaints of floaters in both eyes for 15 days. He was a known case of NHL (Diffuse large B cell lymphoma grade 3) and had completed a full course of chemotherapy and was currently under remission. A complete ophthalmic evaluation was done. His BCVA was 20/30 in the right eye and 20/25 in the left eye. Anterior segment examination showed AC cells 1+ in both eyes. Intraocular pressure in right and left eye was 15 and 14 mm of Hg respectively. The fundus evaluation of the right eye (Fig. 4A) showed vitritis 1+, retinitis lesion at superotemporal arcade along with surrounding few hemorrhages, retinal vasculitis, and the left eye fundus showed retinitis lesions along with hemorrhages in all quadrants and skip vasculitis (Fig. 4B). Therefore a diagnosis of CMV retinitis in both eyes was made and a subsequent vitreous biopsy was done along with intravitreal ganciclovir injection. PCR was positive for CMV and negative for VZV and HSV. His CD4 counts were found to be 186 cells/mm^3^ and he was seronegative for HIV.

The patient was started on the induction phase of Valganciclovir (900 mg, twice daily) and at the end of 3 weeks of induction dose, his retinitis lesion had begun to resolve with central clearing. However, he developed myelosuppression due to Valganciclovir and his absolute neutrophil count dropped to 440/μl. Hence, Valganciclovir was discontinued and he was shifted to weekly intravitreal ganciclovir injections for the maintenance phase. After 2 months of weekly ganciclovir injections, the retinitis lesion had resolved, and hence his treatment was discontinued. However, after 35 days of stopping treatment, he had a recurrence of retinitis in the left eye, and therefore weekly intravitreal ganciclovir injections were reinitiated for the left eye. CD4 count at the time of recurrence was 370 cells/mm^3^. After three months of maintenance regimes of weekly intravitreal ganciclovir injections, they were discontinued. He developed a second recurrence in his left eye 42 days after discontinuation of Ganciclovir. His CD4 counts at second recurrence were 350 cells/mm^3^. He was subsequently maintained on weekly intravitreal Ganciclovir injections.

After 3 months of treatment, intravitreal therapy was discontinued. However he developed a third recurrence after 44 days of stopping the maintenance phase. The CD4 counts were 970 cells/ mm^3^ at the time of his third recurrence. The patient has been now maintained on weekly intravitreal injections (has received a total of 41 injections in the left eye). At the last visit (15 months of follow-up) BCVA in the right and left eyes were 20/200 and 20/400 respectively and fundus evaluation showed optic disc pallor in both eyes (Fig. 4C and D) along with resolved retinitis in the left eye.

## Results

Mean duration of onset of CMV retinitis after initial diagnosis of NHL was 15 months (range 11–20 months). Three patients had a presenting CD4 count above 150 cells/mm^3^ and none of them were below 50 cells/mm^3^. All patients had vitritis and majority of the patients (3 out of 4) had anterior chamber (AC) inflammation. Granular or indolent retinitis (6 out of 7 eyes) was the commonest form of CMV retinitis in patients of NHL and one of the patient (case 1) with granular retinitis later developed fulminant retinitis. Floaters were the commonest presenting complaint. All patients received induction dose of intravitreal ganciclovir (2.5 mg/0.1 ml twice weekly) or Oral Valganciclovir (900 mg twice daily) for 3 weeks after which maintainence regimen was initiated (intravitreal ganciclovir 2.5 mg/0.1 ml once weekly or Oral Valganciclovir 900 mg once daily). Two out of the four patients had a recurrence (mean duration 33.8 days,range 16 days - 44 days) after stopping the maintenance phase of ganciclovir and one patient had significant myelosuppression related to oral valganciclovir which required discontinuation of the drug.

## Discussion

CMV retinitis is an ocular opportunistic infection and a leading cause of ocular morbidity associated with AIDS. It is categorized as an AIDS-defining illness and is usually associated with CD 4 counts < 50 cells/ mm^3^ in patients with HIV [3, 5]. However, apart from AIDS, even malignancies and chemotherapies that target T cells are known to increase the risk of CMV disease [3–5].

CMV retinitis behaves differently in non-HIV patients and can present with increased vitritis and different presentation patterns [9–15].

In our subset of patients, the mean duration between diagnosis of NHL and presentation with CMV retinitis was around 15 months. All patients were non-reactive for HIV. Most of them had bilateral disease (3 out of 4).

Granular or indolent retinitis (6 out of 7 eyes) was the most common form of CMV retinitis in our patients of NHL and was seen in 3 out of the 4 patients. In one of these patients, it evolved into fulminant retinitis later in the course of the disease as seen in another case reported by us previously [12]. Pathanapitoon et al. [13] had retrospectively reviewed the records of 18 HIV-negative patients (22 affected eyes) diagnosed as having posterior uveitis or panuveitis who had aqueous positive for CMV by PCR techniques. The commonest ocular features in their series included focal hemorrhagic retinitis, peripheral retinal necrosis, inflammatory reaction in the anterior segment, vitritis, and retinal vasculitis mostly affecting arteries.

Retinal vasculitis was noted to involve both arteries and veins equally in our patients. Cases 1 and 4 also had associated optic disc pallor (optic neuropathy) at last follow-up which may also be a cause of suboptimal vision.

The most common presenting complaint of these patients was floaters, which was the presenting complaint in 3 out of 4 patients, and this may serve as a clue for underlying vitritis.

In contrast to CMV retinitis in HIV patients where vitritis is less common, NHL patients can present with severe vitritis and panuveitis [10]. All our patients had vitritis and the majority of them (3 out of 4) had AC inflammation (Table 1). The presence of vitritis and retinitis lesions in patients with NHL can lead to a dilemma of an underlying primary intraocular lymphoma (PIOL) and therefore a vitreous biopsy assumes importance in these cases.

**Table 1:** Clinical presentations of CMV retinitis in patients of NHL

	Month and year of presentation	AC reaction/ Vitritis	Duration of onset of CMV after initial diagnosis of NHL	Type of CMV retinitis	CD 4 count at presentation (cells/ mm^3^)	Adverse effects of Valganciclovir	Time for reactivation after stopping maintenance Phase/ CD4 count at the time of recurrence	PCR	Total duration of follow up
**Case 1**	September 2018	AC Cells 1+,Vitritis 1+	12 months	Granular to start and later turned to fulminant	109	Nil	**First recurrence** 16 days CD4 counts - 130 cells/ mm^3^ **Second Recurrence** 32 days CD4 counts 219 cells/mm^3^	CMV	22 months
**Case 2**	October 2019	RE Vitritis 3+	11 months	Granular	278	Nil	on maintenance regimen	CMV	4 months
**Case 3**	June 2015	AC cells 2+,Vitiritis 1+	18 months	Granular	180	N/A	No recurrence	Negative	4 months
**Case 4**	January 2019	AC Cells 1+, Vitritis 1+	20 months	Mixed	186	Neutropenia	**First recurrence** 35 days CD4 counts - 370 cells/ mm^3^ **Second Recurrence** 42 days after stopping maintenance regimen CD4 counts: 350 cells/ mm^3^ **Third Recurrence** 44 days after stopping maintenance regimen CD4 counts: 970 cells/ mm^3^	CMV	15 months

Derzko-Dzulynsky et al. [16] and Chawla et al. [17] had reported a similar diagnostic dilemma in their cases of CMV retinitis in patients with NHL where PCR aided in the diagnosis of intraocular CMV infection. Svozílková P et al. [18] had reported a case wherin simultaneous occurrence of cytomegalovirus retinitis and active intraocular lymphoma was noted. Gooi et al. [19] reported a case of CMV retinitis which mimicked intraocular lymphoma and a retinal biopsy was required for assessment of the final diagnosis.

We had also earlier reported a case of CMV Retinitis in a patient of NHL where one of the differential diagnosis was an intraocular lymphoma [12]. In our series of patients, case 2 had dense vitritis where PIOL was kept as a differential. However, PCR of vitreous biopsy confirmed CMV and ruled out the possibility of PIOL. A vitreous biopsy can therefore be an extremely valuable tool in such situations. In our series one patient (case 3) had PCR negative for CMV however the clinical picture was characteristic for CMV and it responded well to intravitreal ganciclovir injection.

All of our patients had a presenting CD4 count above 100 cells/mm^3^ and none of them were below 50 cells/mm^3^ (the level at which CMV retinitis usually occurs in HIV patients). Occurence of CMV retinitis in patients of NHL with high CD4 counts can be due to exhaustion and senescence of T cells which are major dysfunctional states in the tumor microenvironment [20, 21].

Occasionally a dilemma of Immune recovery uveitis (IRU) can be encountered because of the vitritis. IRU is an important cause of visual morbidity in patients with AIDS. The clinical spectrum of IRU includes vitritis, papillitis, macular edema, and epiretinal membranes [22]. Development of ERM in Case 1 of our series can possibly be due to IRU with high CD4 counts. However, the presence of underlying retinitis in all our cases helped in distinguishing from an IRU even in the presence of vitritis.

Systemic and local treatment including intravenous ganciclovir, intravitreal ganciclovir, and oral valganciclovir remain the mainstay of treatment for CMV retinitis. Valganciclovir is a valyl prodrug of ganciclovir, which inhibits viral DNA polymerase enzyme. It has eight times more bioavailability than ganciclovir and can prevent systemic CMV infection and can also prevent CMV retinitis in the fellow eye. However oral Valganciclovir can be associated with some serious adverse effects including pancytopenia [23]. One patient in our study, (Case 4) had developed severe myelosuppression leading to discontinuation of Valganciclovir. Three of our patients also had relative thrombocytopenia and leucopenia at the initial visit precluding starting valganciclovir at the induction phase however once the blood parameters were stabilized they were started on oral valganciclovir (Cases 1 and 3). It is therefore imperative to check for regular complete blood counts for patients receiving Valganciclovir.

Unlike CMV retinitis in HIV, where we have definite guidelines for discontinuing treatment, [24] discontinuing treatment for CMV retinitis in patients of NHL can be a dilemma. Two of our patients had a reactivation of retinitis after stopping the maintenance phase of ganciclovir and the meantime for it to happen was 33.8 days. Both cases had episodes of recurrence after discontinuing treatment even in the presence of high CD4 counts. CD4 counts therefore cannot be used as a reliable marker as these patients of NHL can have dysfunctional T cells. Case 1 had a CD4 count of 130 cells / mm^3^ at the time of first recurrence and a CD4 count of 219 cells/mm^3^ at the time of the second recurrence. Similarly, Case 4 had three episodes of recurrences after discontinuing treatment. CD4 counts of 370 cells/mm^3^ were noted at the time of the first recurrence and CD4 counts were 350 cells / mm^3^ and 970 cells / mm^3^ at the time of second and third recurrence. In view of the unreliability of CD4 counts and because of multiple recurrences, most of our patients were kept on a long-term maintenance regimen and three patients were receiving treatment even till the last follow-up. There is also a need for regular retinal evaluation of these patients after discontinuation of treatment in view of the risk of recurrences. Hence, larger study is the need of hour to provide a evidence based treatment protocol for CMV retinitis in non HIV patients.

## Conclusion

CMV retinitis in patients with NHL behaves differently from CMV retinitis in HIV patients. Floaters are the most common presenting complaint and indolent or granular retinitis is the most common presentation. Our observations show that this subset of patients can present with AC reaction and vitritis. A vitreous biopsy is extremely valuable in patients with dense vitritis since PIOL can also present with vitritis and retinitis in these cases. CD4 counts are not a reliable indicator and these patients can develop retinitis and recurrences even in presence of high CD4 counts. The mean duration of reactivation of retinitis after stopping the maintenance phase was 33.8 days. Therefore there is a need for a longer duration of treatment and these patients need regular ophthalmic evaluation to detect recurrences.
